# Specialty Grand Challenge for NeuroRehabilitation Research

**DOI:** 10.3389/fneur.2020.00349

**Published:** 2020-05-22

**Authors:** Thomas Platz, Giorgio Sandrini

**Affiliations:** ^1^BDH-Klinik Greifswald, Centre for Neurorehabilitation, Intensive and Ventilation Care, Spinal Cord Injury Unit, University of Greifswald, Greifswald, Germany; ^2^Neurorehabilitation Research Group, University Medical Centre, Greifswald, Germany; ^3^Special Interest Group Clinical Pathways, World Federation for NeuroRehabilitation, North Shields, United Kingdom; ^4^Neurorehabilitation Unit, IRCCS Mondino Foundation, Pavia, Italy; ^5^Department of Brain and Behavioral Sciences, University of Pavia, Pavia, Italy

**Keywords:** rehabilitation, research, evidence, knowledge, guideline

## Global Burden of Disease and Neuro-Disabilities

One of the great challenges the world faces in terms of health care is the increasing number of people living with neuro-disabilities that affect their ability to participate in societal activities. Various neurological conditions such as stroke, multiple sclerosis, or Parkinson's disease, to name just a few, change cognitive, sensory, or motor capacities, alter the emotional well-being of those affected, and lead to disability in their everyday lives.

Over the last few decades, aging populations and reduced mortality in many regions of the world have increased the number of people living with neuro-disabilities considerably, an effect that is still ongoing (1): for 2017, the worldwide prevalence of stroke (thousands) has been estimated to be as high as 104178.7 (95% confidence interval, 95% CI 98454.0–110125.0), and years lived with disabilities (YLD) (counts in thousands) caused by stroke were reported to amount to 18695.4 (95% CI 13,574–23686.9). The stroke-related increase in YLD (percentage change in counts) was 40% (95% CI 38.4–41.4) from 1990 to 2007 and another 43.6% (39.6–47.8) during only 10 years from 2007 to 2017. The numbers are similarly impressive for other neurological disorders (i.e., dementias, Parkinson's disease, epilepsy, multiple sclerosis, motor neuron disease, headache disorders, and others). Taken together, their worldwide prevalence (in thousands) in 2017 was 3121435.3 (95% CI 2951124.5–3316268.0), while YLD (thousands) in 2017 were 3121435.3 (95% CI 2951124.5–3316268.0), with an increase in YLD by 35.1% (95% CI 31.9–38.1) from 1990 to 2007 and by a further 17.8% (95% CI 15.8–20.2) from 2007 to 2017.

These numbers not only demonstrate the huge global burden of disease and prevailing neuro-disabilities, but they indicate a considerable increase in the number of people living with neuro-disabilities with an accelerating dynamic over time (for stroke).

## Clinical Research tO Indicate the Overall Benefit of Neurorehabilitation

Neuro-disabilities cannot be avoided, in spite of great advances that have more recently been achieved in acute medical care. The increase in their prevalence is rather a consequence of more effective health care management, reducing mortality (but not necessarily morbidity), and of aging populations around the globe.

Morbidity and disability are, however, not an inevitable union. Even when organic brain damage cannot be prevented or cured altogether, neurorehabilitation as a specialized form of rehabilitation care can effectively (while most frequently not completely) reduce the burden of disability by promoting functional recovery, compensation of body dysfunction, and/or adaptations, e.g., by the provision of adaptive technology.

Neurorehabilitation is mostly structured as a multi-professional physician-led team approach to health care and has been shown to reduce disability effectively (2).

A Cochrane review with a meta-analysis including 21 randomized controlled trials (RCTs) with a total of 39,994 participants showed a reduced rate of death or institutionalized care (odds ratio, OR 0.78, 95% CI 0.68–0.89) and death or dependence (OR 0.79, 95% CI 0.68–0.90) after multi-disciplinary stroke unit care compared to care in general wards post stroke without significantly increasing length of stay, and independent of age, sex, or stroke severity (3). The situation in low and middle countries (LMIC) with a large diversity of stroke rehabilitation structures sadly supports the notion that adequate rehabilitation efforts effectively reduce disability (4): with better structure and processes of care such as the availability of multi-disciplinary stroke care units, patients were more likely to be alive, independent, and living at home 1 year after stroke; absence of rehabilitation, on the other hand, was associated with a higher level of disability.

## A Complex Pattern of Research is Required to Promote Neurorehabilitation as a Medical Specialty

Neurorehabilitation is a medical discipline that, for its scientific advancement, necessitates a complex pattern of research. Brain functions and their dysfunctions are complex issues, as are any interventions that intend to promote functional recovery after brain damage and hence to improve brain function. Such interventions target specific brain network activities and functions and can include training procedures (“therapy”), electrical or magnetic stimulation of the brain or body limbs, and medication targeting the brain and its transmitter systems.

“From bench to bedside” involves a multitude of research avenues for neurorehabilitation: basic research, translational research, clinical trials (pilot and confirmatory), collating evidence across clinical trials and providing an evidence synthesis by systematic reviews and meta-analyses, the systematic generation of evidence-based practice guidelines, and finally their regional adaption into clinical pathways (5). All of these research areas need to be addressed for such diverse neurological conditions as stroke, multiple sclerosis, or Parkinson's disease, with their distinct neuropathologies and different patterns of cognitive, sensory, and motor dysfunctions as well as emotional disorders.

In addition, there is a great need to perform research from a global health perspective. Technologies that generate a clinical benefit in neurorehabilitation, e.g., arm rehabilitation robots (6), electromechanical gait training (7), virtual reality applications (8), tele-rehabilitation (9), or non-invasive brain stimulation (10), might be considered candidates for an adaptation for low- and middle-income countries (LMIC); low-cost technologies could be developed for a broader international distribution and clinically evaluated.

Furthermore, priority research is necessary to elaborate rehabilitative needs and therapeutic options when new challenges like the current novel coronavirus (2019-nCoV) pandemic manifest themselves. Most people affected by the Coronavirus Disease 2019 (COVID-19) have mild symptoms and recover, while 6.1% become critically ill (respiratory failure, septic shock, and/or multiple organ dysfunction/failure) (11) and might develop a post-intensive care syndrome, PICS, with motor, cognitive, and emotional disorders necessitating intensive rehabilitation (12, 13). Research has to document the epidemiology and rehabilitation needs of COVID-19 cases and their clinical course. It should further address the effectiveness of neurorehabilitation treatment including the use of new technologies for home care purposes (e.g., use of low-cost technologies such as smartphones or tablets for virtual medical examination, counseling, and tele-rehabilitation), as well as health care system questions (e.g., how rapidly increasing demands for services should be coped with), and guidance (practice recommendations).

## The Continuum of Care in Neurorehabilitation and Its Research

Another specific aspect of neurorehabilitation for people with neuro-disabilities is that we do not have a single “phase” of disease and do not need to take care of people affected at a given point in time only when the disease becomes evident. On the contrary, the care of people with neuro-disabilities frequently involves a lifetime perspective.

For example, for people with stroke, it is well-understood that the best outcome is achieved with a multi-stage rehabilitation pathway (14–16). Such a dedicated pathway starts with acute rehabilitation and post-acute rehabilitation (usually inpatient services), and continues with out-patient rehabilitation, home-based rehabilitation, community-based rehabilitation, and long-term and sustained rehabilitation.

Accordingly, research and knowledge management in neurorehabilitation need to take the continuum of care for people with neuro-disabilities into consideration.

## The Need for Education and Knowledge Dissemination

Neurorehabilitation teams frequently include physicians, physiotherapists, occupational therapists, speech and language therapists, psychologists, nurses, and social workers trained in neurorehabilitation as a “core set” of involved disciplines. The reason is two-fold. For one, all of their specialized professional knowledge and therapeutic skills are essential to treat people with neuro-disabilities. Secondly, it is the team approach itself that contributes essentially to the overall clinical benefit and not just the availability of diverse professions, each working on its own (2, 3).

These affordances can, however, not be met in many regions of the world, especially in many low- and middle-income countries (LMIC). There is a substantial lack in the number of health care professionals for rehabilitation in LMICs, and, frequently, the types of health care professionals needed for rehabilitation teams are not at all available. A few examples (17): high-income countries have, on average, more than 900 physiotherapists per million inhabitants; the corresponding number is <10 physiotherapists in many countries in Sub-Saharan Africa and the South-East Asia Region. Further, high-income countries have more than 300 speech and language therapists per 1 million inhabitants, while some low-income countries in the African region have no speech and language therapists for the entire population.

There is thus a huge demand for education in neurorehabilitation. The need includes (a) the establishment of qualifying program for various disciplines in many countries, (b) specialized training in neurorehabilitation for health care professionals holding their basic professional qualification (physicians and allied health professionals), (c) continued medical education for those who have received specialized training, and (d) fast knowledge distribution in new challenging situations or “game-changing” opportunities for clinical practice.

Initiatives to address these needs are far from being sufficient. An example for (b) is the core curriculum for neurorehabilitation developed by the European Federation for Neurorehabilitation (18), and an example for (c) are the summer schools on neurorehabilitation organized by the Word Federation for NeuroRehabilitation (19). For the transnational harmonization of education initiatives, it could be useful to start in countries with similar health care systems (e.g., in Europe) while being accessible for international attendees.

## Knowledge Management Platforms as Key Structures

**Figure 1:**
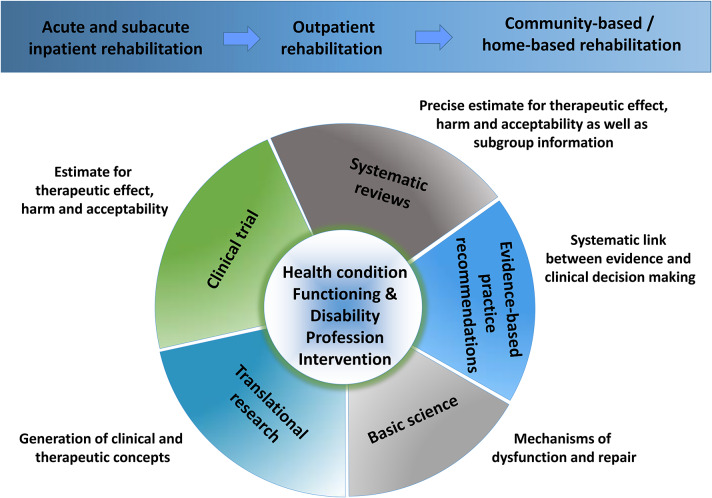
Neurorehabilitation research perspectives. The figure shows the complexity of the background to research in neurorehabilitation. The complexity is caused by the multitude of health conditions, alterations of functioning and disabilities, and of respective interventions as provided by different health care professions along a continuum of health care from acute rehabilitation to community-based rehabilitation. The diversity of research focuses is further enlarged by a necessity to engage in both basic and translational research, clinical trials, and synthesis of the evidence in systematic reviews with meta-analyses and by a methodologically sound link from evidence to the clinical decision via evidence-based practice recommendations.

With all the complexity of neurorehabilitation in terms of the diversity of health conditions leading to neuro-disabilities, the research avenues involved, the multitude of healthcare professions and settings from inpatient to community rehabilitation (compare Figure 1), and the lack of human resources and knowledge hubs in many regions of the world, there is a great need for knowledge management platforms that host high-quality up-to-date research across this wide spectrum and make that knowledge publically available, not only to those who can afford to pay for it but especially to those who are put at a disadvantage both by their limited regional professional resources and by any financially restricted access to high-quality professional knowledge sources.

Such platforms provide an opportunity to advance the science in the field by providing a possibility to collate and synthesize research knowledge across the boundaries of individual research cluster and professions (20, 21) as well as for various health care situations, be it in high- or low- and middle-income countries.

## Conclusions

*Frontiers in Neurology* is a leading journal in its field, publishing rigorously peer-reviewed articles across a wide spectrum of basic, translational, and clinical research that help improve patient care. Its *Neurorehabilitation section* provides an interdisciplinary platform for new developments in this highly complex field that demands the involvement of a broad range of professionals and to create a forum for the exchange of knowledge among these different specialists.

The *Neurorehabilitation section* focuses primarily on clinical studies, though it also attracts papers dealing with basic and translational research relevant to clarifying mechanisms or scientifically addressing new therapeutic concepts for neurorehabilitation. Systematic reviews that synthesize evidence from clinical practice and provide more precise estimates for the evaluation of benefit-risk ratios and the acceptability of interventions together with subgroup information are highly welcome, as are reviews that systematically link evidence syntheses to evidence-based practice recommendations. In addition, the section wants to promote scientific exchange for the adaptation of therapeutic concepts and technology to the diverse health care backgrounds that exist at an international level.

The section equally wants to promote health care in neurorehabilitation by serving as a platform for *Research Topics* with the collation of research papers on topics of great interest to the scientific and/or clinical community.

Taken together, the *Neurorehabilitation section*, which is driven by academic standards and makes its publications freely available for a worldwide readership, makes a significant contribution to quality in neurorehabilitation research and healthcare with a global perspective for the ultimate sake of those affected by neuro-disabilities and in need of the best possible professional help.

## Author Contributions

TP designed and wrote the manuscript, and GS revised it critically for intellectual content.

## Conflict of Interest

The authors declare that the research was conducted in the absence of any commercial or financial relationships that could be construed as a potential conflict of interest.
